# A comparison of total thoracoscopic versus robotic approach for cardiac myxoma resection: a single-center retrospective study

**DOI:** 10.1007/s11701-023-01531-z

**Published:** 2023-01-17

**Authors:** Yanyi Liu, Zhuang Liu, Xin Li, Yiyao Jiang, Chenghao Lu, Chengxin Zhang, Shenglin Ge

**Affiliations:** 1 Present Address: Department of Cardiac Surgery, The First Affiliated Hospital of Anhui Medical University, No.218, Jixi Road, Hefei, 230022 Anhui China; 2 Present Address: Department of Cardiac Surgery, The First Affiliated Hospital of Bengbu Medical College, Bengbu, 233000 Anhui China

**Keywords:** Myxoma, Minimally invasive surgical procedures, Thoracoscopy, Robotics

## Abstract

Advances in instrumentation and technique have facilitated minimally invasive surgeries for cardiac myxoma treatment. This study aims to compare the clinical outcomes between the thoracoscopic and robotic approaches for myxoma resection. Intraoperative data and postoperative data of 46 patients who underwent either thoracoscopic (*n* = 15) or robotic (*n* = 31) cardiac myxoma resection in our center between July 2013 and September 2022 were retrospectively compared. There was no in-hospital death in either group. Meanwhile, the operative time and cardiopulmonary bypass time were significantly shorter in the robotic group than in thoracoscopic group (*P* = 0.015 and *P* = 0.035, respectively). Furthermore, shorter ICU stays (*P* = 0.006), shorter postoperative mechanical ventilation time (*P* = 0.035) and less thoracic drainage (*P* = 0.040) were observed in the robotic group. However, the operating room costs and total hospital costs were both significantly lower in thoracoscopic group (*P* = 0.004 and *P* = 0.007, respectively). There was no significant difference between two groups regarding the incidence of postoperative complications (*P* > 0.05). Lastly, a faster return to exercise was noted in robotic group than in thoracoscopic group (Log-Rank *χ*^2^ = 4.094, *P* = 0.043). Both approaches can be safe and feasible for myxoma resection. However, regardless of higher expenses, the robotic myxoma resection approach provides shorter operation time, less postoperative thoracic drainage, and faster recovery than total thoracoscopic technique.

## Introduction

Cardiac myxoma is the most common type of primary cardiac tumor [1], which accounts for about 75–80% of all the primary neoplasms in the heart [2]. The prevalence of the cardiac myxoma is estimated to be 0.5 per million per year [3]. Once diagnosed, surgical resection is usually recommended to prevent fatal complications such as valvular obstruction and embolization [4]. Since the first successful removal of left atrial myxoma in 1954 [5], sternotomy has been widely accepted as the standard procedure for cardiac myxoma resection [3]. However, minimally invasive techniques such as total thoracoscopic and robotic surgical approaches have evolved considerably during the last decade and obtained excellent outcomes [6, 7]. Thus far, the application of these minimally invasive techniques has expanded to mitral valve surgeries, congenital heart disease repair, atrial fibrillation ablation, coronary artery bypass grafting (CABG), and cardiac myxoma resection [8–11]. Nevertheless, compared with other diseases, reports on myxoma resection using either the robotic or total thoracoscopic technique is still relatively scarce, mainly due to the low incidence of myxoma and the limited number of hospitals equipped with the robotic system or thoracoscopic device [4].

Both the thoracoscopic and robotic approaches for myxoma resection have been associated with better visualization, smaller incision, and faster recovery than conventional sternotomy [12, 13]. Wei et al. previously compared the clinical outcomes between thoracoscopic and robotic surgeries for mitral valve repair [8]. They concluded that the thoracoscopic technique has more advantages in postoperative recovery and hospitalization expenses. However, research comparing the thoracoscopic and robotic procedures for myxoma resection has not been reported in any previous literature. Therefore, in this study, we sought to compare the clinical outcomes between these two approaches for myxoma resection. To the best of our knowledge, this study is the first of its kind reported in the literature.

## Methods

### Patient selection

A total of 46 patients who underwent minimally invasive cardiac myxoma resection in our center from July 2013 to September 2022 were included. Among these, 31 patients were treated using the Da Vinci Surgical System (robotic group), including 29 patients with left atrial myxoma (LAM) and 2 patients with right atrial myxoma (RAM). The other 15 cases were treated using a thoracoscopic procedure (thoracoscopic group), including 14 patients with LAM and 1 patient with RAM. Preoperative transthoracic echocardiography (TTE) was performed for each patient to evaluate the size, location, attached sites, and obstruction status of the tumors. The selection criteria for both the total thoracoscopic and robotic procedures were as follows: (1) without the requirement for concurrent procedures such as coronary surgery, aortic procedures, or aortic valve surgery; (2) without history of right thoracotomy, severe pericarditis, or severe pleuritis; (3)without thorax deformity or morbid obesity; (4) without peripheral vascular problems which may restrict femoral cannulation; (5) without history of asthma or chronic obstructive pulmonary disease (COPD). Furthermore, postoperative pathological examination of all subjects confirmed that all excised tumors were cardiac myxomas.

### Robotic surgery technique

After double-lumen endotracheal intubation under general anesthesia, a transesophageal echocardiography (TEE) evaluation was performed. The patients were then maintained in supine position with the right thorax elevated to 30°. The cardiopulmonary bypass (CPB) was achieved via right internal jugular venous cannulation, right femoral arterial and venous cannulation. After the initiation of single-lung ventilation, a 3-cm working port was made on the anterior axillary line in the fourth intercostal space (ICS). The camera was inserted anteriorly at the same ICS. Two 8-mm endoscopic trocars were then placed at the anterior axillary line in the 3rd and 6th ICS for robotic arms. Meanwhile, the atrial retractor was inserted through the 5th ICS at the mid-clavicular line. After docking the Da Vinci robot (Intuitive Surgical, Sunnyvale, CA, USA), the pericardium was incised. Superior and inferior vena cava were sequentially blocked following the initiation of CPB. Cases with right atrial myxoma were directly excised via right atriotomy on the beating heart. For cases with left atrial myxoma resection, aortic blocking was completed using a Chitwood cross-clamp inserted through the 3rd or 4th ICS at the right mid-axillary line, while antegrade perfusion of cold-blood cardioplegic solution was administered. All the myxomas were excised completely. The defect of the atrial septum or atrial wall after myxoma resection was repaired by continuous suturing or using a pericardial patch when needed. TEE was required at the end of the procedure to assess if there was a residual tumor or interatrial shunting after septal reconstruction.

### Thoracoscopic surgery technique

**Fig. 1:**
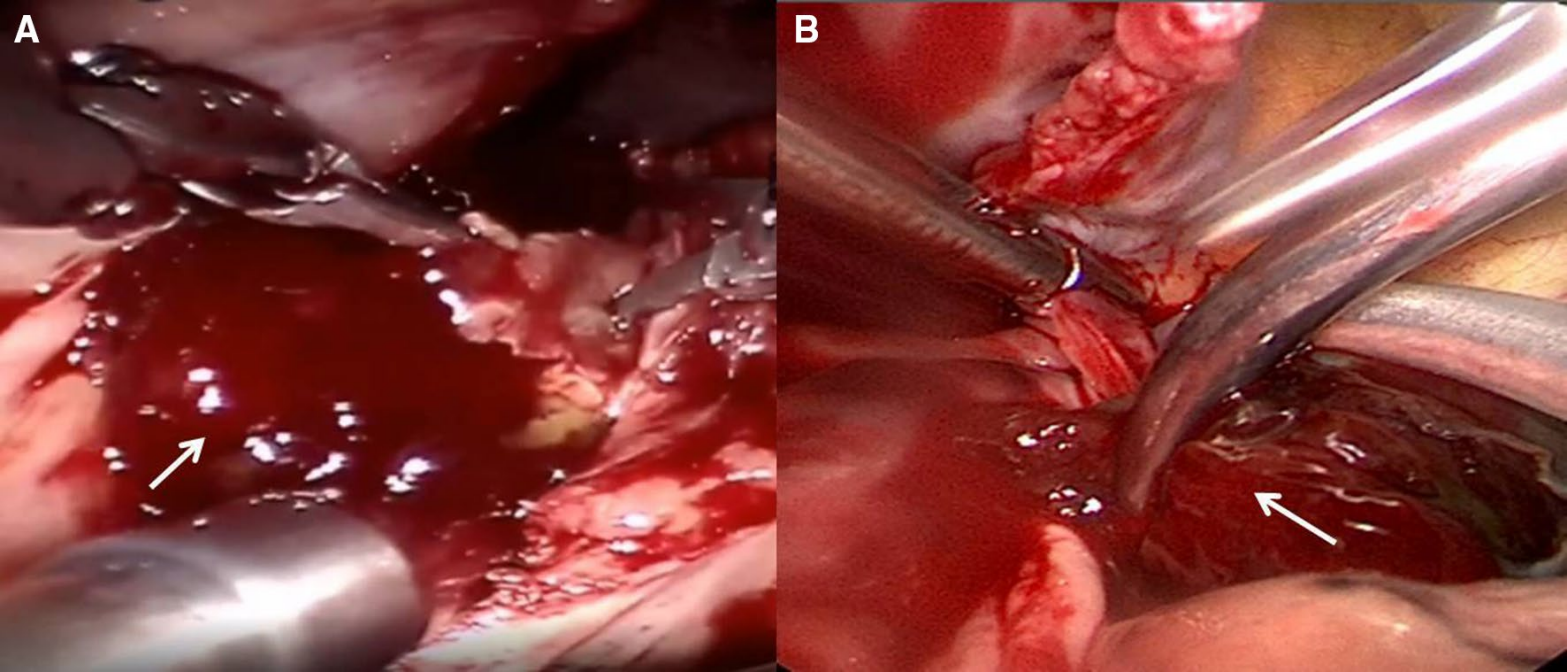
Operative scene of robotic and thoracoscopic surgery. **A** Resection of left atrial myxoma (with arrow) during robotic surgery. **B** Resection of right atrial myxoma (with arrow) during thoracoscopic surgery

Three ports were made on the right chest wall. Port 1 (3–4 cm) was made at the 4th ICS of the right anterior axillary line, where the thoracoscope was inserted. Port 2 (2–3 cm) was made parasternally in the 2nd or 3rd ICS for entries of left-hand instruments. Port 3 (2–3 cm) was made in the 5th or 6th ICS lateral to the mid-clavicular line for entries of right-hand instruments. The establishment of the peripheral CPB and intra-thoracic part of the operation were the same as those of the robotic group. The operative scene of thoracoscopic and robotic myxoma resection is presented in Fig. 1.

### Data collection and follow-up

The operative parameters, in-hospital outcomes, and follow-up results between the robotic and thoracoscopic groups were compared. Peak pain score (Visual Analogue Scale, VAS) after surgery was observed to assess the postoperative pain. Clinical and follow-up data were obtained from electronic medical records and follow-up surveys. The patients were followed up at the outpatient center after discharge. A telephone interview was required for those who were lost to the outpatient center. In the follow-up, the time to return to exercise and the occurrence of any major adverse cardiovascular and cerebrovascular events (MACCE) were investigated for both groups. MACCE was defined as major adverse cardiovascular and cerebrovascular events since the day of operation, including events of cardiac death, acute coronary syndrome, new-onset arrhythmia, stroke, and peripheral vascular embolism.

### Statistical analysis

Statistical analysis was performed using SPSS 25.0 (IBM, Chicago, Illinois, USA). Continuous variables with normal distribution were expressed as mean ± standard deviation (SD), and were compared by the Student’s *t*-test. Continuous variables with skewed distribution were expressed as median and 25–75 percentile, and were compared using the Mann–Whitney *U* test. Categorical variables were described as numbers and percentages, and were compared by Pearson’s Chi-square test, continuity adjusted Chi-square test or Fisher’s exact test. Survival data were analyzed using the Kaplan–Meier method and compared by log-rank test. A *p*-value of < 0.05 was considered statistically significant.

## Results

There was no significant difference between two groups in terms of sex, age, BMI, cardiac function, blood creatinine, and parameters of preoperative echocardiography (Table 1). The incidence rates of various comorbidities, including coronary artery disease (CAD), diabetes, hypertension, embolism, and preoperative atrial fibrillation, were also similar between the two groups (Table 1).

**Table 1:** Baseline characteristics

	Thoracoscopic	Robotic	*P*-value
	(*N* = 15)	(*N* = 31)	
Gender (male/female)	5/10	13/18	0.575
Age (years)	54.13 ± 11.26	53.10 ± 15.16	0.815
BMI (kg/m^2^)	22.74 ± 2.82	21.55 ± 3.10	0.219
LVEF (%)	63.73 ± 4.76	63.42 ± 3.34	0.797
LAD (mm)	40.00 ± 6.32	39.36 ± 6.12	0.744
LVEDD (mm)	47.71 ± 5.11	47.62 ± 3.69	0.944
PASP (mmHg)	35.0 (27.0,51.0)	32.0 (28.0,46.0)	0.851
Blood creatinine (µmol/L)	61.15 ± 16.12	59.10 ± 13.24	0.650
Cardiac function [*n *(%)]			0.933
NHYA I/II	9 (60.0)	19 (61.3)	
NHYA III/IV	6 (40.0)	12 (38.7)	
Hypertension [*n *(%)]	2 (13.3)	3 (9.7)	0.709
Diabetes [*n *(%)]	0 (0.0)	2 (6.5)	1.000
Coronary artery disease [*n *(%)]	0 (0.0)	1 (3.2)	1.000
Embolism			
Cerebral embolism [*n *(%)]	2(13.3)	6 (19.4)	0.928
Peripheral embolism [*n *(%)]	0 (0.0)	0 (0.0)	NA
Atrial fibrillation [*n *(%)]	0 (0.0)	0 (0.0)	NA

*BMI* body mass index, *LVEF* left ventricular ejection fraction, *LAD* left atrial diameter, *LVDD* left ventricular end-diastolic diameter, *PASP* pulmonary artery systolic pressure, *NYHA* New York Heart Association functional class

The surgical data are presented in Table 2. All 46 operations were completed without transition to median sternotomy in either group. However, shorter CPB time (97.90 ± 24.48 vs. 118.27 ± 38.82 min, *P* = 0.035) and shorter operation time (225.39 ± 33.83 vs. 297.13 ± 98.70, *P* = 0.015) were observed in robotic group. Left atrial myxomas were more often excised via left atriotomy or biatriotomy when performing the robotic procedure. In contrast, left atrial myxomas were more frequently resected via right atriotomy when performing the thoracoscopic procedure (*P* < 0.05). The myxomas excised in the thoracoscopic group were 41.33 ± 15.52 mm long and 31.27 ± 12.83 mm wide on average, while the myxomas excised in the robotic group were 47.94 ± 16.04 mm long and 34.90 ± 12.75 mm wide in average. However, there was no significant difference regarding myxoma size between the two groups (*P* > 0.05). Aortic clamping time, concomitant procedures, and surgical timing were also similar between the two groups (*P* > 0.05).

**Table 2:** Surgical data

	Thoracoscopic	Robotic	*P*-value
	(*N* = 15)	(*N* = 31)	
Operation time (min)	297.13 ± 98.70	225.39 ± 33.83	0.015
CPB time (min)	118.27 ± 38.82	97.90 ± 24.48	0.035
Aortic clamping time (min)	70.0 (51.0,78.0)	55.0 (44.0,69.5)	0.124
Myxoma size			
Length (mm)	41.33 ± 15.52	47.94 ± 16.04	0.193
Width (mm)	31.27 ± 12.83	34.90 ± 12.75	0.370
Myxoma location			
Left atrium [*n *(%)]	14 (93.3)	29 (93.5)	1.000
Right atrium [*n *(%)]	1 (6.7)	2 (6.5)	1.000
Approach to left-sided myxoma			0.017
Left atriotomy [*n *(%)]	8 (53.3)	21 (67.7)	
Right atriotomy and trans-septal incision [*n *(%)]	4 (26.7)	0 (0.0)	
Biatriotomy [*n *(%)]	2 (13.3)	8 (25.8)	
Concomitant procedures			
Mitral annuloplasty [*n *(%)]	1 (6.7)	5 (16.1)	0.670
Tricuspid annuloplasty [*n *(%)]	3 (20.0)	9 (29.0)	0.767
Surgical timing [*n *(%)]			1.000
Emergent	1 (6.7)	2 (6.5)	
Elective	14 (93.3)	29 (93.5)	

*CPB* cardiopulmonary bypass

Perioperative outcomes are shown in Table 3. Significantly shorter ICU stay time, shorter postoperative ventilation time, and less postoperative drainage were observed in robotic group (*P* < 0.05).Furthermore, patients in robotic group appears to leave bed earlier than thoracoscopic group (*P* = 0.001). However, no significant difference was observed regarding postoperative peak pain score between two groups (*P* = 0.760). Despite the less intraoperative RBC usage in robotic group (*P* < 0.05), there was no significant difference in terms of total RBC usage and transfusion rate between two groups (*P* > 0.05). Operating room costs and total hospital costs of robotic group were both significantly greater than those of thoracoscopic group (*P* < 0.05).

**Table 3:** Perioperative outcomes

	Thoracoscopic	Robotic	*P*-value
	(*N* = 15)	(*N* = 31)	
Mechanical ventilation time (h)	13.0 (5.0, 19.0)	6.0 (3.0, 15.0)	0.035
ICU length of stay (h)	39.0 (38.0, 44.0)	20.0 (19.0, 41.0)	0.006
Postoperative hospital stay (d)	9.0 (8.0, 13.0)	9.0 (8.0, 11.0)	0.422
Thoracic drainage^a^ (ml)	190.0 (105.0, 290.0)	110.0 (60.0, 215.0)	0.040
Intraoperative RBC transfusion (U)	0.0 (0.0, 2.5)	0.0 (0.0, 0.0)	0.034
Postoperative RBC transfusion (U)	1.5 (0.0, 3.5)	2.0 (0.0, 4.0)	0.468
Total RBC transfusion [U, median (U)]	3.5 (0.0, 4.5)	3.0 (0.0, 4.0)	0.725
Transfusion rate (all blood product), *n *(%)	10 (66.7)	20 (64.5)	0.886
Postoperative leaving-bed time (d)	5.0 (4.0, 7.0)	3.0 (2.0, 5.0)	0.001
Peak pain score^b^	4.93 ± 2.19	4.71 ± 2.37	0.760
Total hospital costs (× 10^4^$)	1.13 (0.92, 1.35)	1.39 (1.21, 1.67)	0.007
Operating room costs (× 10^4^$)	0.10 (0.08, 0.11)	0.11 (0.10, 0.38)	0.004

*ICU* intensive care unit

^a^The first 24-h thoracic drainage after surgery

^b^Maximum score of VAS (Visual Analogue Scale) after surgery

The postoperative in-hospital complications are listed in Table 4. There was no in-hospital mortality in either group. There was also no significant difference between two groups in the incidence of various postoperative complications, including new-onset atrial fibrillation, stroke, peripheral embolism, delirium, delayed mechanical ventilation, acute renal failure, pneumonia, subcutaneous emphysema, and wound infection (*P* > 0.05).

**Table 4:** Postoperative complications

	Thoracoscopic (*N* = 15)	Robotic (*N* = 31)	*P*-value
30-day mortality [*n *(%)]	0 (0.0)	0 (0.0)	–
New-onset atrial fibrillation [*n *(%)]	2 (13.3)	6 (19.4)	0.928
New-onset systemic embolism [*n *(%)]			
Stroke [*n *(%)]	0 (0.0)	1 (3.2)	1.000
Peripheral embolism [*n *(%)]	1 (6.7)	0 (0.0)	0.326
Delirium [*n *(%)]	1 (6.7)	2 (6.5)	1.000
Reoperation for bleeding [*n *(%)]	0 (0.0)	0 (0.0)	–
DMV [*n *(%)]	1 (6.7)	0 (0.0)	0.326
Acute renal failure [*n *(%)]	1 (6.7)	0 (0.0)	0.326
Pneumonia [*n *(%)]	0 (0.0)	2 (6.5)	1.000
Subcutaneous emphysema [*n *(%)]	2 (13.3)	2 (6.5)	0.827
Wound infection [*n *(%)]	1 (1.0)	0 (0.0)	0.326

*DMV* delayed mechanical ventilation (> 24 h)

**Fig. 2:**
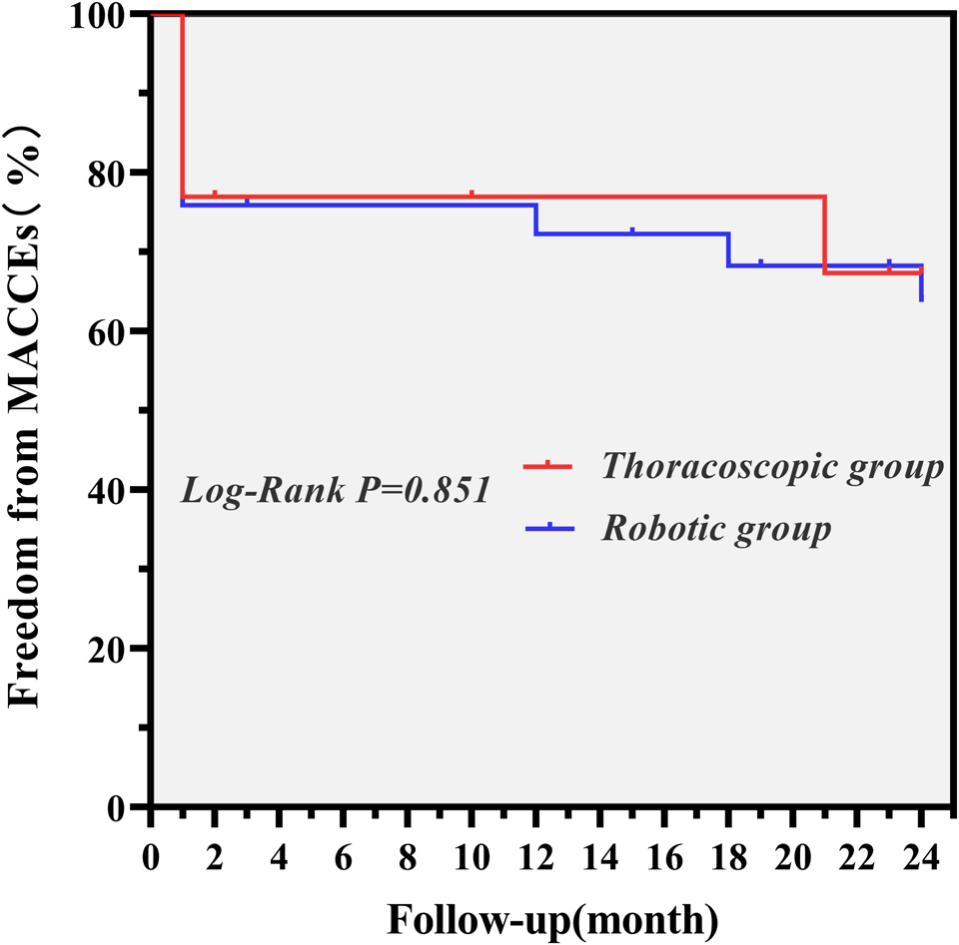
The Kaplan–Meier curve for MACCE

**Fig. 3:**
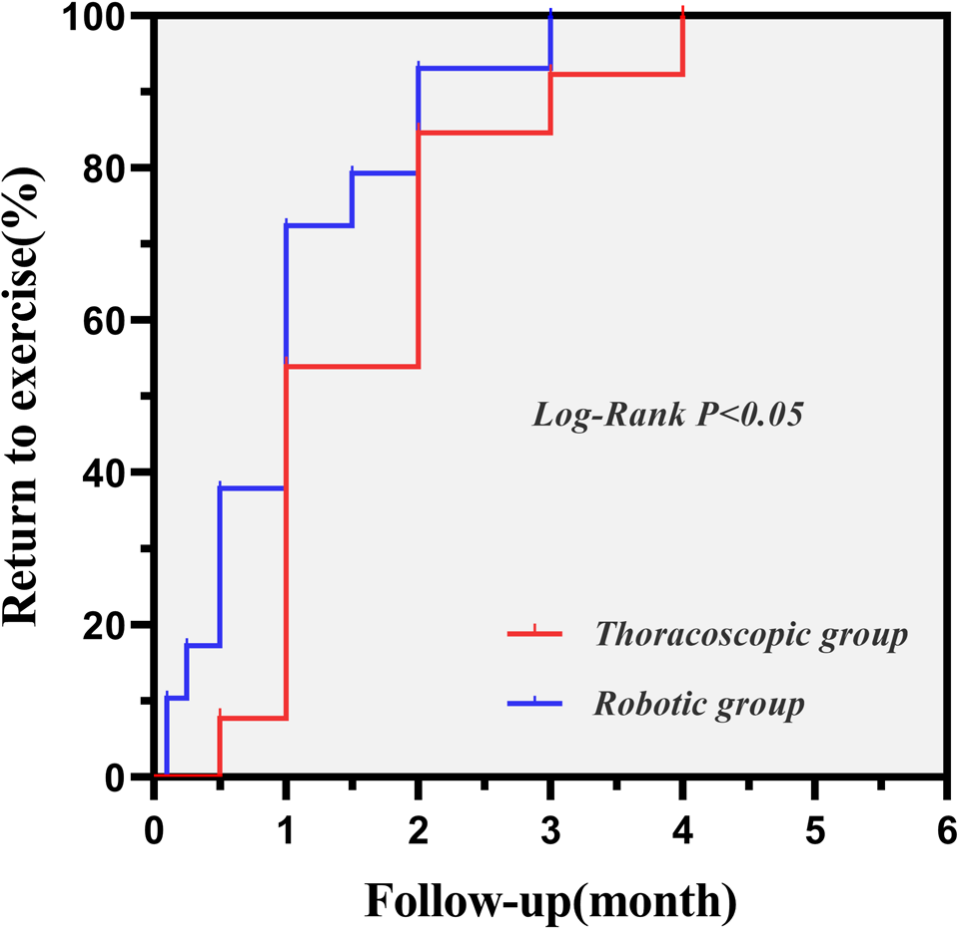
Kaplan–Meier analysis on the rate of returning to exercise

13 patients (13/15, 86.67%) in the thoracoscopic group were followed for 2–65 months (mean 34.69 ± 16.74 months; median 35.0 months), while 29 patients (29/31, 93.55%) in the robotic group were followed for 3–62 months (mean 35.72 ± 18.55 months; median 37.0 months). There was also no follow-up death in either group. During follow-up, one patient of thoracoscopic group presented with acute coronary syndrome 21 months after the operation. Meanwhile, three patients of robotic group presented with new-onset atrial fibrillation 12, 18, and 24 months after surgeries, respectively. Overall, the freedom from MACCE was 76.9, 76.9, and 67.3% at 6 months, 1 year, and 2 years after thoracoscopic surgeries, respectively. However, the 6-month, 1-year, and 2-year freedom from MACCE for patients who underwent robotic surgeries were 75.9, 72.2, and 63.7%, respectively. No significant difference was noted in freedom rate from MACCE between groups (Log-Rank *χ*^2^ = 0.035, *P* = 0.851, Fig. 2). The mean time to return to exercise was 1.65 ± 0.99 months in the thoracoscopic group, and 1.06 ± 0.79 months in the robotic group. A faster return to exercise was noted in robotic group than in thoracoscopic group (Log-Rank *χ*^2^ = 4.094, *P* = 0.043, Fig. 3).

## Discussion

Since the first successful robotic cardiac myxoma resection performed by Murphy et al. in 2005 [14], the procedure has been demonstrated as a safe and feasible method with rapid recovery, cosmetic superiority, and improved postoperative quality of life when compared to the conventional sternotomy approach [4, 5, 13, 15–18]. This study aims to explore the differences in clinical outcomes between robotic and thoracoscopic myxoma resection, which are not well documented in previous literature. In our study, both the CPB time and operation time were found to be significantly shorter in the robotic group. We speculated that this difference might result from the fact that the wrist-like articulated instruments of robotic system can move at six degrees of freedom without tremors. In addition, this can help improves the speed and precision of surgical manipulation, thus shortening the CPB and operation time. In comparison, the long-shafted thoracoscopic instruments, to some extent, limits the surgeon’s mobility and can easily cause fatigue. In addition, the process of careful hemostasis is also essential for an uneventful postoperative course. Potential bleeding sites in myxoma resection surgeries include the atriotomy incision, the aortic purse for the cardioplegia needle, and the port incision [4]. Robotic system can provide maximum visualization of bleeding points and intracardiac structure using three-dimension (3D) high-definition imaging [19], which greatly facilitates surgical hemostasis intraoperatively. Significantly, less 24-h postoperative drainage of robotic group was observed in this study, reflecting a better hemostatic effect and less tissue damage. In this study, no conversion to sternotomy was observed in either group, indicating the feasibility of both approaches for myxoma resection.

The current study is the first to compare the postoperative parameters between robotic and thoracoscopic approaches to myxoma resection. In previous studies comparing the robotic and thoracoscopic approaches in mitral valve repair, the ICU duration and postoperative ventilation time were reported to be longer in the robotic group [8]. In contrast, our study found that durations of ICU stay, bed stay, and postoperative mechanical ventilation were significantly lower in the robotic group, which indicates a faster recovery using the robotic technique in myxoma resection. In addition, the robotic group also showed a significantly faster return to exercise after discharge.

Massive allogenic blood transfusion is an independent risk factor for nosocomial pneumonia following cardiac surgeries and plays an important role in postoperative kidney injury [16]. Wei S et al. [8] concluded that thoracoscopic surgeries have a lower transfusion rate than robotic surgeries (52.2% vs. 64.5%) [8]. We found a different trend in the transfusion rates in this study (64.5% for robotic, 66.7% for thoracoscopic); however, no significant difference was observed between the two groups. Besides, there was no significant difference in the total perioperative RBC transfusion between the two groups, whereas the robotic group seemed to receive less intraoperative RBC transfusion.

Cardiac myxoma resection via sternotomy had an early postoperative mortality of 0% to 10% based on previous studies [17, 20–22]. In contrast, no 30-day mortality was observed for either group in this study. This also indicates that both of these two minimally invasive approaches were safe and feasible. Balkhy et al. reported that atrial fibrillation was the most common complication following robotic cardiac surgeries, with an incidence of 12% [23]. Similarly, new-onset atrial fibrillation was also the most frequent complication in this study (19.4% for robotic, 13.3% for thoracoscopic). However, the incidence of atrial fibrillation after robotic surgeries was higher than previously published data [13, 23]. This may be partly due to the limited sample size and a higher proportion of biatriotomy in the robotic group. Although biatriotomy can provide convenience for a large-sized myxoma excision [24], this technique is assumed to be the potential cause of atrial fibrillation after myxoma resection surgery [4, 25]. In this study, up to 25.8% of patients in the robotic group underwent left atrial myxoma resection via biatriotomy, and we speculated that this could be the reason for the high incidence of new-onset atrial fibrillation after robotic surgeries. New-onset stroke rate was low in both study groups (0% for thoracoscopic, 3.2% for robotic). We suggested that the magnified visualization provided by the robotic or thoracoscopic approaches has improved the surgeon’s ability to recognize fragments shed by friable myxoma, which in turn has helped to prevent postoperative stroke. Furthermore, no significant difference was noted between the two groups in terms of in-hospital complications rate and incidence of MACCEs. However, our study has unsurprisingly confirmed that the robotic group involves more costs in comparison with the thoracoscopic group. This particular disadvantage of high costs may limit the application of the robotic technique to some degree. Thus, the thoracoscopic technique could be an effective alternative when the robotic technique is not affordable.

## Limitations

First, our study is a non-randomized, retrospective study. We recognize that a selection bias may exist that cannot be fully eliminated. Thus, the lack of randomized and prospective design is an inherent limitation. Second, this study only reported a single-centered experience with limited sample size, which may reduce the power to detect significant differences. A multi-center study with larger sample size and longer follow-up time are still required. Furthermore, the costs cited in our study may not be reproduced in other countries due to the factors such as the inflation rate and different economic levels.

## Conclusion

This study demonstrates that both thoracoscopic and robotic approaches are safe and feasible for cardiac myxoma resection. However, regardless of higher expenses, robotic myxoma resection can offer shorter operation-related time, less postoperative thoracic drainage, and faster recovery than total thoracoscopic technique.

## Data Availability

The clinical data used to support the findings of this study are available from the corresponding author upon reasonable request.
